# Using network dynamic fMRI for detection of epileptogenic foci

**DOI:** 10.1186/s12883-015-0514-y

**Published:** 2015-12-21

**Authors:** Sanja Nedic, Steven M. Stufflebeam, Carlo Rondinoni, Tonicarlo R. Velasco, Antonio C. dos Santos, Joao P. Leite, Ana C. Gargaro, Lilianne R. Mujica-Parodi, Jaime S. Ide

**Affiliations:** Department of Biomedical Engineering, Stony Brook University School of Medicine, Stony Brook, NY 11794 USA; Department of Radiology, A. A. Martinos Center for Biomedical Imaging, Massachusetts General Hospital, Charlestown, MA 02129 USA; Department of Neurosciences and Behavior, University of Sao Paulo (USP), Ribeirao Preto, SP 14049 Brazil; Department of Science and Technology, Federal University of Sao Paulo, Sao Jose dos Campos, SP 12231 Brazil

**Keywords:** Epilepsy, Seizure, fMRI, Power spectrum scale invariance, Autocorrelation function, Classification, Complexity, Chaos, Fractal

## Abstract

**Background:**

Epilepsy is one of the most prevalent neurological disorders. It remains medically intractable for about one-third of patients with focal epilepsy, for whom precise localization of the epileptogenic zone responsible for seizure initiation may be critical for successful surgery. Existing fMRI literature points to widespread network disturbances in functional connectivity. Per previous scalp and intracranial EEG studies and consistent with excessive local synchronization during interictal discharges, we hypothesized that, relative to same regions in healthy controls, epileptogenic foci would exhibit less chaotic dynamics, identifiable via entropic analyses of resting state fMRI time series.

**Methods:**

In order to first validate this hypothesis on a cohort of patients with known ground truth, here we test individuals with well-defined epileptogenic foci (left mesial temporal lobe epilepsy). We analyzed voxel-wise resting-state fMRI time-series using the autocorrelation function (ACF), an entropic measure of regulation and feedback, and performed follow-up seed-to-voxel functional connectivity analysis. Disruptions in connectivity of the region exhibiting abnormal dynamics were examined in relation to duration of epilepsy and patients’ cognitive performance using a delayed verbal memory recall task.

**Results:**

ACF analysis revealed constrained (less chaotic) functional dynamics in left temporal lobe epilepsy patients, primarily localized to ipsilateral temporal pole, proximal to presumed focal points. Autocorrelation decay rates differentiated, with 100 % accuracy, between patients and healthy controls on a subject-by-subject basis within a leave-one-subject out classification framework. Regions identified via ACF analysis formed a less efficient network in patients, as compared to controls. Constrained dynamics were linked with locally increased and long-range decreased connectivity that, in turn, correlated significantly with impaired memory (local left temporal connectivity) and epilepsy duration (left temporal – posterior cingulate cortex connectivity).

**Conclusions:**

Our current results suggest that data driven functional MRI methods that target network dynamics hold promise in providing clinically valuable tools for identification of epileptic regions.

## Background

Epilepsy is one of the most prevalent neurological disorders, affecting approximately 50 million people worldwide [1]. It is characterized by seizures, resulting from abnormal transient change in the synchronized firing of neurons [2]. For one-third of patients with focal epilepsy, debilitating seizures persist despite antiepileptic drug therapy, leaving surgical resection of the suspected epileptogenic focal region as the most effective treatment option [3, 4]. Precise localization of the epileptogenic zone responsible for initiation and propagation of seizures, and its delineation from eloquent cortex, are crucial for successful surgery. However, current standard non-invasive surgical evaluation (looking for congruence of seizure semiology, abnormalities on structural MRI images and spikes in scalp EEG recordings) fails to identify an epileptic focus in approximately 40 % of patients with drug resistant epilepsy [5]. The current gold standard for localization of focal regions includes identification of an epileptogenic zone on intracranial EEG recordings combined with postoperative seizure freedom following its resection; however, invasive pre-surgical workup carries additional risks and has been associated with complications in about 23 % of patients [5]. Thus, the development of noninvasive techniques capable of accurately localizing epileptogenic regions on a subject-by-subject basis will be critical for improving surgical outcomes.

Multimodal studies, especially those utilizing simultaneous EEG-fMRI recordings, increasingly have been used to provide complementary information in presurgical work-up. EEG-fMRI allows mapping of hemodynamic changes related to seizure-related events, such as interictal discharges (IEDs). Spikes are manually detected in EEG data and, in traditional “spike-correlated” analysis, they are treated as zero-duration events, convolved with canonical hemodynamic response function (HRF), and included as regressors of interest in a General Linear Model (GLM) along with simultaneously acquired BOLD time series as dependent variables. fMRI maps related to IEDs often show multiple regions or “networks,” rather than focal singularities, and thus effective connectivity approaches such as Dynamic Causal Modeling (DCM) have been proposed to identify which brain regions drive the generation of seizures within the epileptic network [6, 7].

The EEG-fMRI approach suffers from a few potential drawbacks. First, a significant portion of subjects do not experience enough detectable IEDs during simultaneous recording. This problem is partially addressed by introduction of topography-related techniques, which, instead of requiring simultaneously recorded spikes, use subject-specific voltage maps based on long-term video monitoring [8]. Second, epilepsy may alter the shape of the HRF, which could result in decreased sensitivity [9]. Third, in addition to setup time, EEG-fMRI requires on average 30 min of motionless cooperation from subjects, which may be problematic for certain patients, especially children. Fourth, the use of scalp EEG is inherently constrained by its limited sensitivity to deep activity. Despite this, sensitivity of topography-related EEG-fMRI in refractory focal epilepsy was found to be about 80 % [8, 10]. However, measured sensitivity appears to be highly study-dependent. For example, a recent study proposing concurrent use of four different modalities found that topography-related EEG-fMRI method on its own showed clinically meaningful result in five out of twelve studied patients ([11]). Here we propose an alternative data-driven method utilizing only resting state fMRI data.

As a matter of general research strategy, any method with potential to identify seizure onset zones first needs to be validated with respect to a “ground truth.” In the case of epilepsy, the most straightforward option is to use patients with clinically well-defined focal regions; after validation against these cases, one can then apply the method to more challenging cryptogenic cases. Mesial Temporal Lobe Epilepsy (MTLE) is the most prevalent and best-characterized subset of drug resistant focal epilepsy in adults, and thus is an ideal cohort for validation of novel methods aimed at identification and localization of epileptic foci and/or networks.

Brain regions involved in the onset and propagation of MTLE have been studied extensively, with the most commonly associated pathology being hippocampal sclerosis (HS). Seizures originating in the medial temporal region are known to rapidly spread to lateral temporal regions, the insula, the thalamus, and the contralateral temporal lobe (among other regions) [12], suggesting that MTLE may be characterized by a network disturbance. Indeed, recent structural and functional MRI studies have revealed widespread abnormalities, with structural changes primarily involving atrophy of ipsilateral temporal pole and other temporolimbic structures [13, 14]. Functional MRI (fMRI) studies have found changes in functional connectivity of the temporal region with other brain areas, along with impaired resting state networks such as perceptual, attention and default mode networks [15–24].

Graph theoretical network analyses of brain networks use sets of nodes connected by edges in order to quantify general structural features of brain connectivity between regions. Numerous studies suggest that healthy brain networks show a high degree of *small-worldness* (for a review see [25]), which provides a balance between local clustering (characteristic of highly ordered, *regular* networks) and long-range connections (characteristic of low order, *random* networks). This balance reflects the result of synaptic optimization over the efficiency of information transfer and the need for redundancy in case of injury. In individuals with focal temporal and extratemporal neocortical epilepsy, fMRI, EEG, MEG, and intracerebral recordings suggest that this balance appears to be shifted towards predominantly local clustering [20, 26, 27], which may predispose the network towards synchronized oscillations characteristic of seizures [28].

However, findings are not clear-cut. While many studies report decreases in functional connectivity localized near the suspected seizure onset zone [16, 17, 22, 23], others show increases [15, 20, 29–31], potentially due to differences in connectivity metrics used, as well as heterogeneity of patient populations and small sample sizes. It is important to note that, in spite of the benefits of considering epileptic networks, accurate localization is still clinically desirable as suggested by the relatively high success rate of surgical treatment for ‘focal’ epilepsies (about 66 %) [2]. Yet most fMRI studies aimed at localization of the epileptogenic zone point to widespread abnormalities that are, at best, lateralized to one of the hemispheres.

Complex systems produce outputs that are balanced between overly chaotic and overly predictable dynamics; as such, they have the advantage of requiring a minimum of energy both to respond to inputs as well as to return to baseline (as required for allostasis). Complexity in brain activity has been observed and modeled on many levels, from neurotransmitter release [32], neuronal spiking [33, 34] and local field potentials [35] to slow cortical potentials [36], electrocorticography (ECoG) [37] and EEG [38], suggesting that scale-free behavior may be fundamental to efficient neural information processing. Deviations from optimal range of functioning in fMRI time series have been used diagnostically in identifying brain-based disease [37, 39, 40]. In the case of a disease as heterogeneous as epilepsy, fMRI’s exploratory capability (simultaneously acquiring functional information over focal, hemispheric, and whole-brain neural networks) may provide a clinically valuable tool in guiding placement of intracranial EEG, as the process of seizure generation is not necessarily confined to a focal area and may involve distant or contralateral areas of the brain. Here we propose to use autocorrelation function (ACF) as a measure of complexity in resting-state fMRI time series, with the aim of localizing deviations from optimal dynamics in epilepsy.

Brain trauma increases risk for seizures. One possible mechanism suggested by animal models of epilepsy is the enhanced synaptic sprouting due to MMP-9 mediated matrix-degradation that occurs as a compensatory response to injury [41]. Alternatively, metabolic damage affecting the brain's ability to utilize glucose may impact the brain’s creation of long-range, but not short-range, connections, upsetting balance between the two. Thus, we hypothesized that the hippocampal sclerosis common to MTLE patients might result in hubs surrounded by more dense local connectivity. This can constrain dynamics within the hub either by increased density of inhibitory or excitatory inputs, both of which would result in less chaotic resting state fMRI time series. We expected abnormal dynamics to be identifiable through comparison of decay rates of the time series’ ACF in patients and in healthy controls. This is consistent with previous findings from scalp and intracranial EEG studies [42–45], as well as with excessive local synchronization during interictal discharges.

Less chaotic dynamics in focal region(s) may lead to increased local synchronization and transient seizures. Repeated seizures could in turn lead to damage in connections of this region with contralateral regions and major hubs in the brain. We therefore hypothesized that these disconnections would be detectable via seed-to-voxel functional connectivity analyses of fMRI time-series, using the region with abnormal dynamics (identified through ACF analyses) as a seed. Finally, we expected that abnormalities in connectivity would correlate with the duration of epilepsy as well as with patients’ cognitive performance on a verbal memory recall task. To determine whether group differences achieving statistical significance continued to hold on the single-subject level (as required for future application of individual neurodiagnostics to other, cryptogenic, forms of epilepsy), we applied Gaussian Process Classification with leave-one-subject-out cross validation to ACF decay rates in regions with altered dynamics.

## Methods

### Participants

Nineteen patients (mean age = 40 years ± 13; 8 males, 11 females) with mesial left temporal lobe epilepsy (LTLE) were included in this study based on a clear clinical diagnosis of unilateral (left) temporal epileptic activity according to concordant clinical information. The inclusion criteria were: 1) seizure semiology consistent with MTLE, which included epigastric, autonomic, or psychic auras followed by behavioral arrest, progressive clouding of consciousness, oroalimentary and manual automatisms, and autonomic phenomena; 2) anterior and mesial temporal interictal spikes; 3) video-EEG monitoring with seizure onsets arising exclusively from the temporal lobe; 4) MRI with no other lesion than hippocampal atrophy and a hyperintense signal on T2-weighted sequences; and 5) medically refractory MTLE, defined as failure to respond to at least two antiepileptic drugs after adequate trials. For patients whose scalp ictal EEG recordings were inconclusive, foramen oval electrodes or depth electrodes were used to define lateralization of seizure foci. Table 1 lists demographic and clinical characteristics of all patients studied here. Nineteen healthy controls (mean age = 41 years ± 12; 8 males, 11 females), age and sex-matched to patients (2-sample *t* test *p* = 0.78), were scanned under the same protocol. Because temporal lobe epilepsy affects the hippocampus, and is associated with progressive memory deficits [46], in order to assess clinical symptoms patients were asked to complete the Logical Memory Delayed Recall (LM-DR) subtest of the Wechsler Memory Scale – Revised [47]. This task requires subjects to recall specific details of information presented orally in a story format thirty minutes after a single exposure. The research protocol was approved by the local Ethical Committee of Clinics Hospital at Ribeirao Preto, São Paulo, Brazil. Written informed consent was obtained from the patient for publication of their individual details in this manuscript. All subjects were older than 18 years of age and were capable of providing informed consent.

**Table 1:** Demographic and clinical information

Patient	Gender	Age/Onset	Frequency (month)	Febrile seizures	Seizure type	Interictal EEG	Ictal EEG	MRI findings	Antiepileptic medications	Surgical outcome
1	F	44/18	30	No	Masticatory automatisms	Left temporal paroxysms	Bilateral theta rhythm, late left lateralization	Left HA	PHT, TPM	3
2	M	59/10	4	Yes	Complex partial	100 % left temporal	Left temporal	Left HA	CBZ	1
3	F	27/13	4	No	Complex partial	70 % left, 30 % right temporal	Left temporal	Left HA	LTG, CLB, OXC	1
4	M	47/24	4	No	Lack of consciousness, no aura	Left temporal sharp waves (89 %, T1, T9)	Left temporal theta	Left HA	CBZ, LTG, CLB	1
5	F	32/19	0.5	No	Complex partial	Rare left temporal	Left temporal	Discrete left HA	CBZ, PB, CLB	1
6	F	34/25	4	No	Complex partial	53 % left, 47 % right temporal	Left temporal	Discrete left HA, normal volume	TPM, OXC, CLB	3
7	M	26/5	4	No	Complex partial	Normal	Left temporal delta rhythm	Left HA	VPA, OXC, CLB	1
8	F	44/9	4	No	Complex partial	70 % left, 30 % right temporal	Left temporal	Left HA	CBZ, TPM, CLN	3
9	F	47/17	4	No	Lack of consciousness, automatisms	Slow theta in left temporal	Bilateral theta activity followed by left temporal theta	Left HA	LTG, CBZ	1
10	M	37/2	2	No	Complex partial	95 % left, 5 % right temporal	Left temporal	Left HA	OXC, LTG	1
11	M	46/18	n/a	No	Complex partial	Slow waves in left temporal	Left hemisphere diffuse desynchronization	Left HA	PHT, PB	1
12	F	52/14	2	Yes	Complex partial	100 % left temporal	Left temporal	Left HA	CBZ, TPM	2
13	F	22/19	3	Yes	Tonic-clonic secondarily generalized	Left spikes	Left temporal	Left HA	OXC, VPA, CLB	1
14	F	22/1	16	Yes	Complex partial	Left temporal	Left temporal	Left HA	TPM, OXC	1
15	F	49/15	1	No	Complex partial	100 % left temporal	Left temporal	Left HA	CBZ, PB	1
16	M	59/53	1	No	Complex partial	100 % left temporal	Left temporal	Left HA	PHT	3
17	M	20/4	30	Yes	Epigastric aura and lack of consciousness	Left temporal slow waves	Left temporal	Left HA	CBZ, VPA	1
18	F	30/10	30	No	Complex partial seizures preceded by sensitive auras	Left temporal theta rhythm	Complex partial seizure	Discrete HA + double cortex	LTG, CLB	n/a
19	M	19/8	8	No	Complex partial	100 % left temporal	Left temporal	Left HA	TPM, CBZ, CLN	3

*Abbreviations*: *HA* hippocampal atrophy, *PHT* Phenytoin, *TPM* Topiramate, *CBZ* Carbamazepine, *LTG* Lamotrigine, *CLB* Clobazam, *OXC* Oxcarbazepine, *PB* Phenobarbital, *VPA* Valproic Acid, *CLN* Clobazam. Surgical Outcome: 1 - seizure free, 2 - significant improvement, 3 - no improvement

### Data acquisition

All subjects were scanned using a Philips Achieva 3 T scanner with an eight-channel head coil. Whole brain functional volumes were acquired under resting state using soft-tone sequences (TR = 2000 ms, TE = 30 ms, 200 volumes, voxel dimensions: 3 × 3 × 4 mm, 32 slices, 0.5 mm gap, matrix size = 80 × 80, flip angle = 80°). Subjects were instructed to keep their eyes open and to refrain from falling asleep. The eyes open condition was used rather than eyes closed condition to ensure that subjects remained awake during scanning. T1-weighted anatomical images were acquired using a conventional 3D-T1 MPRAGE sequence (TR = 7.0, TE = 3.2, matrix size = 240 × 240, flip angle = 8°, 1 mm isotropic voxels).

### fMRI data preprocessing

Functional MRI data were preprocessed by correcting for motion (rigid realignment, 6°-of-freedom), slice-time correction, normalization to MNI space (affine registration followed by a nonlinear transformation between average fMRI and EPI template, and sync interpolation), and smoothing with an 8-mm FWHM Gaussian kernel in SPM8 (www.fil.ion.ucl.ac.u/spm).

### Autocorrelation function (ACF) method

An autocorrelation function (ACF) measures similarity (cross-correlation) of a signal with itself over different time lags, and can therefore be used to identify shifts towards chaos vs. order in the time domain, in a manner that is analogous to power spectral scale invariance (PSSI) analysis [40, 48, 49] in the frequency domain. PSSI and ACF are related via the Wiener-Khinchin theorem, which states that Fourier transform of the ACF is the power spectral density (*P*(*f*) = ∫_− ∞_^∞^*ACF*(*t*)*e*^− 2*πift*^*dt*).

PSSI of fMRI time series has already been used to quantify limbic dysregulation in trait anxious adults [49] and effectively discriminate between normal and pathogenic network dynamics in schizophrenia [40] and generalized anxiety disorder (DeDora et al., under review). PSSI analysis is based on the finding that fMRI BOLD time series exhibit power spectral density that follows a power law [50] *P*(*f*) ~ 1/*f*^*β*^, where β is the scaling exponent. The scaling exponent β then measures relative frequency content of the signal and is evaluated as the negative slope of a straight line fit to power spectral density as a function of frequency on log-log scale. Linear least squares method is employed. In contrast, the ACF of voxel-wise BOLD time series can be modeled as an exponential decay:

$$ ACF(t)=a{e}^{-bt}, $$

for which *t* is time lag in units of TR, and *a* and *b* are constants such that *b* > 0. The rate of this decay, which is proportional to the constant *b*, may be used as a compact measure of randomness of a time series, with higher *b* signifying faster decay (more randomness) and lower *b* signifying slower decay (more persistence). ACF b values are then related to the mean lifetime decay of a signal, τ (seconds), via the relation τ = 1/b × TR. Conceptually, τ represents the length of time that a signal maintains a high degree of association with its past values. A random time series (white noise) would have τ = 0. Larger values of τ would imply longer memory in the signal.

ACF has several advantages as compared to PSSI. First, ACF decay times have physiologically intuitive meaning, understood as self-similarity over different lengths of time (in seconds). Second, ACF model fits for fMRI time-series are improved compared with PSSI, because ACF avoids the need to use linear least squares fitting in log-log space [51].

Prior to whole brain voxel-wise computation of ACF b values, to remove potential effects of nuisance variables and improve signal to noise ratio, we performed further preprocessing of fMRI time series. The preprocessing procedure included detrending and regression of mean white matter and mean cerebrospinal fluid (CSF) signals, as well as regression of six degrees of motion. Motion parameters were derived from the realignment procedure, while global regressors were obtained from canonical masks for white matter and CSF included in the SPM8. To rule out head motion related artifacts on b values, we confirmed that there were no statistically significant differences in movement between the two subject groups using root mean square displacement (2 sample *t*-test: *p* = 0.37). Root mean square displacement was calculated as the square root of the sum of squares of all six motion parameters [52].

The residual time series were band-pass filtered in the 0.01 - 0.1 Hz frequency range using 10th order Butterworth filter. Fitting was performed within a frequency range of 0.01-0.1 Hz since low frequency oscillations in this band show power-law behavior [37, 50], making PSSI-related inferences valid in this range, and are furthermore of special interest in resting-state fMRI connectivity analyses since they have been shown to have neuronal basis [51, 53, 54].

We estimated voxel-wise time series ACF using the Econometrics Toolbox implemented in MATLAB R2010a. We then fit an exponential function of the form *y* = *ae*^− *bx*^ to the first 9 (lag = 8) points of each voxel’s ACF using the nonlinear least-squares fitting method as implemented in the Curve Fitting Toolbox, also part of MATLAB R2010a. Lag of 8 (16.8 s at TR of 2 s) was determined as most appropriate based on considerations of goodness of fit of the exponential function and duration of canonical hemodynamic response [55].

Group differences in voxel-wise *b* values were examined using a two sample *t*-test in SPM8. Clusters greater than 26 voxels with *p* < 0.005 were isolated and used in further analyses. Prior to voxel-wise t-tests, the Lilliefors goodness-of-fit test (as implemented in REST toolbox [56]) was used to confirm that voxel-wise ACF-b values were normally distributed within each group of subjects. We found that the hypothesis of normality was supported by the vast majority (94 %) of brain voxels in each group at the *p* = 0.05 level.

### Classification

To verify that the group differences in b values were statistically significant and capable of differentiating between patients and healthy controls, even on an individual subject-by-subject basis, we conducted follow-up machine learning analyses on ACF b maps using Pattern Recognition for Neuroimaging Toolbox (PRoNTo) [57]. We constrained the analyses to those regions that were identified as having different b values between the two groups using the 2-sample *t*-test in SPM8. We employed Binary Gaussian Process Classification with leave one subject out cross-validation. Follow-up permutation testing (with 2000 repetitions) was used to test whether the obtained overall and class accuracies were significantly above chance, and thus indicate whether the pattern of ACF decay times in those brain regions encodes sufficient information to diagnose (correctly classify) MTLE.

### Functional connectivity

**Fig. 1:**
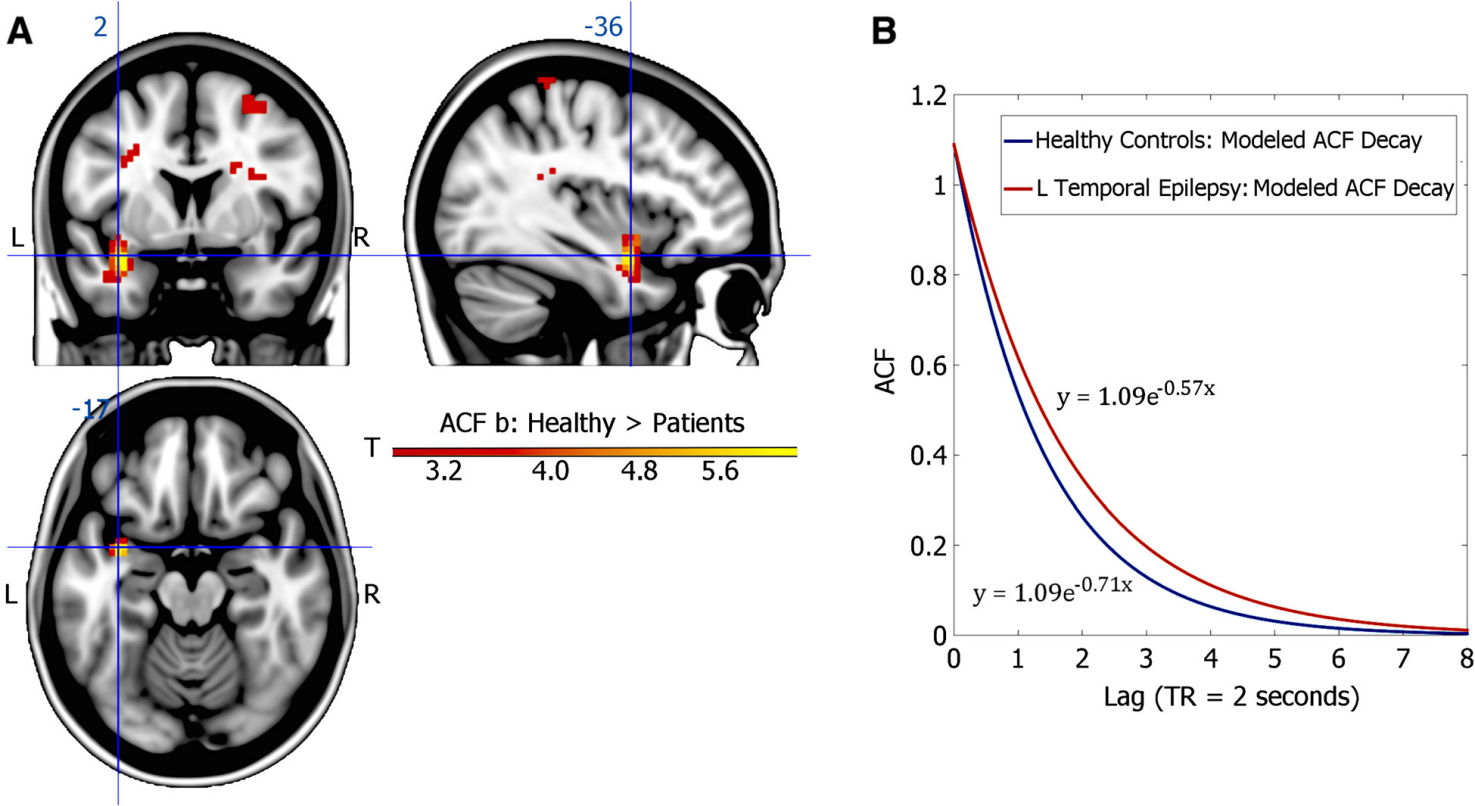
*Autocorrelation decay rates (ACF b) are altered close to presumed focal regions*. **a**: Left Temporal Lobe Epilepsy (LTLE) patients exhibit lower ACF b values (slower decay of autocorrelation) relative to Healthy Controls (HC) in the Left Superior Temporal Pole (peak MNI coordinate [−36, 2, −17], cluster extent = 59 voxels, peak *p* = 1.18 × 10^−8^, *b*_HC_(cluster) = 0.71 ± 0.09, *b*_LTLE_(cluster) = 0.57 ± 0.05)). **b**: Modeled ACF decay for the left superior temporal pole cluster for HC and LTLE patients

In order to gain further understanding of the functional network features that might underlie abnormalities in fMRI signal dynamics, we used the cluster in the left anterior temporal pole identified as having most significantly different ACF b values in patients compared to controls (cluster extent = 59 voxels; Fig. 1a) as a seed in seed-to-voxel functional connectivity analysis using CONN Toolbox (http://www.nitrc.org/projects/conn/; v 13.p). We examined the differences in seed-to-voxel connectivity between LTLE patients and healthy controls using one-sided 2 sample t-tests. Time series were extracted from spatially smoothed images. They were detrended, despiked (a hyperbolic tangent squashing function was applied to reduce the influence of potential outlier scans), and white matter, cerebrospinal fluid, and motion parameters from SPM’s realignment step and their first derivatives were regressed out as confounds. Finally, the residual time series were band-pass filtered in the [0.01-0.1] Hz range. Correlation maps were computed from the seed to all voxels in the brain and transformed to Fisher-z values (to ensure normality) prior to performing t-tests to look at differences between the two groups. We identified clusters of voxels that were significantly differently connected to the region exhibiting constrained dynamics (identified via ACF analysis) in patients relative to controls. We then used correlation analysis to test whether connectivity of these clusters to the seed was related to duration of epilepsy and verbal memory as measured by the LM-DR task.

### Graph-theoretic analyses

To determine whether complexity and connectivity results reflected global differences in network structure, we used the CONN Toolbox to investigate graph-theoretic characteristics of the network with nodes at clusters identified as having different ACF b values in patients compared to controls. Bivariate correlation of the mean time series was used as a measure of association between the nodes. For this 16-node network, we compared the two subject groups with respect to Global Efficiency, Local Efficiency, Betweenness-Centrality, Average Path Length, Clustering Coefficient, and Degree under a range of values of cost (0.1 – 0.3) [58, 59].

## Results

### Left temporal lobe epilepsy patients show constrained dynamics of left temporal pole

Unbiased (exploratory) analyses identified the *superior left temporal pole* (peak MNI coordinate: (−36 2–17), *p* < 0.005 uncorrected, cluster extent = 59 voxels, peak T_36_ = 7.11; Fig. 1) as showing the greatest differences between LTLE patients and healthy controls; patients showed significantly decreased ACF b values (i.e., slower ACF decay, more constrained dynamics). In addition, 15 other regions, all exceeding 27 voxels, showed lowered ACF b values in patients at *p* < 0.005. Regions involved include portions of the *right inferior frontal gyrus*, *left thalamus*, *right precuneus*, *right caudate*, *bilateral heschl gyrus*, *bilateral inferior parietal and postcentral area*, and *bilateral insula*. These results are presented in Table 2. No regions showed increased ACF b values in patients compared to controls. We repeated the 2-sample *t*-test 38 times, each time leaving one subject out. Each time, a cluster at MNI (−36 2–17) was found to be significantly different between the LTLE patients and healthy controls, confirming reliability across subjects, and the absence of outlier-effects. In addition, to probe the potential for single-subject analyses, we compared voxel-wise whole-brain individual patients’ ACF b maps to the average healthy control ACF b map, and found that for 13 out of 19 patients there was a cluster (>6 voxels, median size = 32 voxels) at the identical coordinate (−36 2–17) which had b values that were more than two standard deviations lower than those in the same region in the mean control map. Remaining patients had such clusters within the left temporal lobe.

**Table 2:** List of regions showing significantly different values of ACF b in patients relative to controls

	Left TLE < healthy controls			
	Peak MNI	Location description (AAL)	Cluster extent	*t*-test (peak-level T (p))
1	[−36, 2, −17]	L Superior Temporal Pole, Middle Temporal Pole, Insula, Amygdala	59	7.11 (1.18 × 10^−8^)
2	[13, 23, 42]	R Inferior Frontal Gyrus, Pars Triangularis, Inferior Frontal Gyrus, Pars Opercularis	71	4.59 (2.61 × 10^−5^)
3	[−27, −7, 28]	L White Matter (Precentral)	43	4.57 (2.79 × 10^−5^)
4	[−12, −10, −2]	L Thalamus	33	4.56 (2.85 × 10^−5^)
5	[−45, −37, 43]	L Inferior Parietal Lobule,	69	4.40 (4.64 × 10^−5^)
		Postcentral Gyrus		
6	[8, 21, 25]	R Caudate	69	4.31 (5.95 × 10^−5^)
7	[36, −22, 19]	R Insula, R Heschl	27	4.20 (8.37 × 10^−5^)
8	[30, −43, 52]	R Inferior Parietal Lobule	88	4.10 (1.13 × 10^−4^)
9	[15, −46, 7]	R Precuneus	34	4.07 (1.22 × 10^−4^)
10	[−24, −34, 70]	L Postcentral Gyrus,	60	3.99 (1.53 × 10^−4^)
		Paracentral Gyrus		
11	[30, 41, 1]	R White Matter (Frontal)	42	3.89 (2.01 × 10^−4^)
12	[12, −112, −2]	L Calcarine	31	3.84 (2.41 × 10^−4^)
13	[−30, −43, 25]	L White Matter (Parietal)	31	3.68 (3.78 × 10^−4^)
14	[30, 2, 58]	R Middle Frontal Gyrus,	29	3.62 (4.46 × 10^−4^)
		Superior Frontal Gyrus		
15	[−54, −10, 10]	L Heschl	29	3.46 (6.95 × 10^−4^)
16	[3, −91, 10]	L Calcarine	30	3.28 (1.20 × 10^−3^)

Two-sample *t*-test was performed in SPM8 (*p* < 0.005, k ≥ 27)

### Classification analyses achieve 100 % accuracy in distinguishing patients vs. controls

**Fig. 2:**
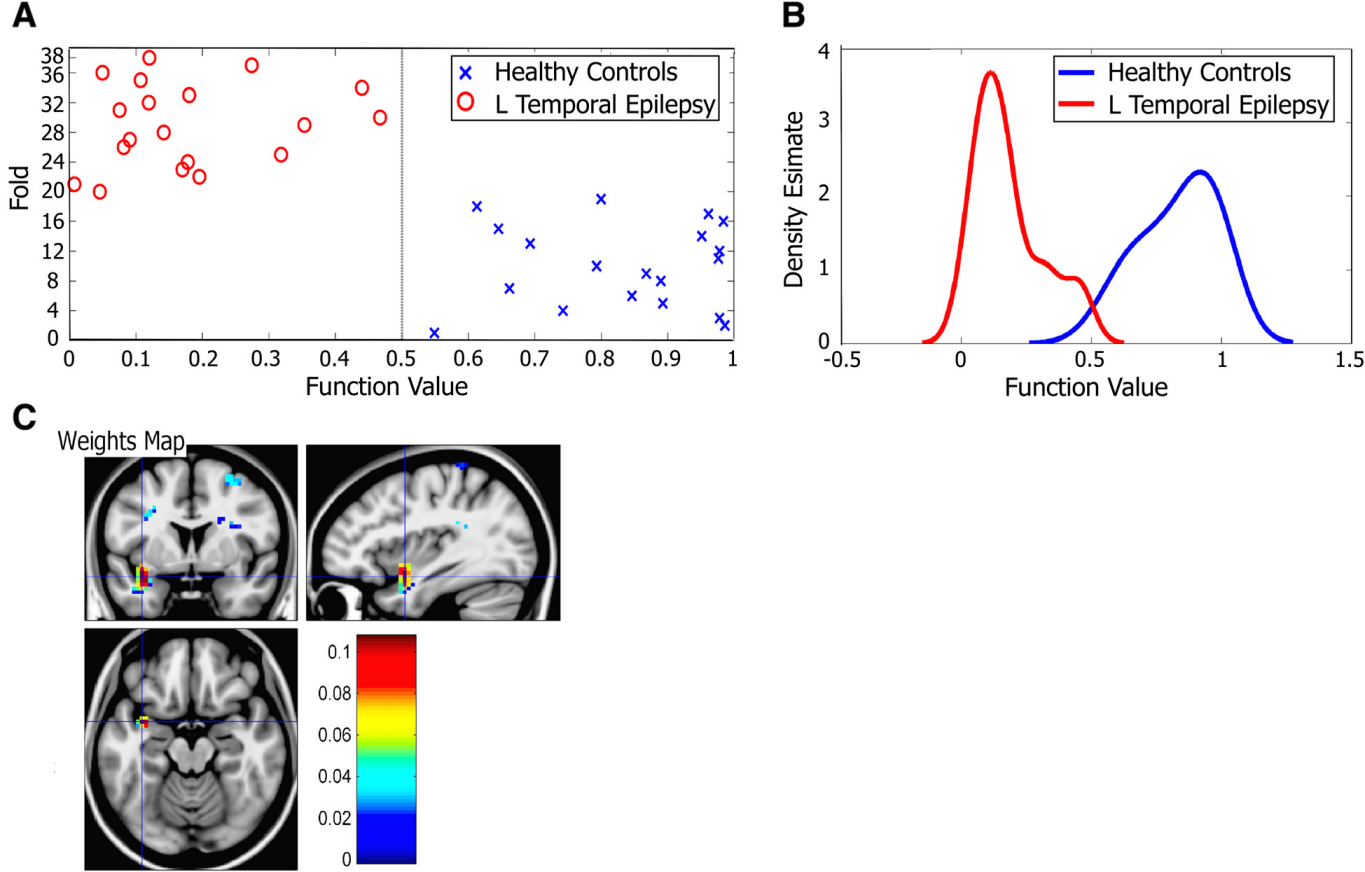
*Autocorrelation decay rates classify patients with epilepsy with 100 % accuracy*. **a**: Plot of prediction values at each level of the leave-one-subject-out cross-validation (fold) shows 100 % accuracy when employing Gaussian Process Classification within Pattern Recognition for Neuroimaging Toolbox (PRoNTo) and using group differences in ACF b values as a 2^nd^ level mask. **b**: Density estimates of predicted function values for the two classes have a small overlap area, which is a sign of a good classifier. **c**: Weights map shows that the left temporal lobe cluster carries most weight in the decision process

To verify that the differences in ACF b values were not only statistically significant at the group level, but also capable of differentiating between patients and healthy controls even on a subject-by-subject basis, we conducted follow-up classification analyses on ACF b maps using Gaussian Process Classification with leave one subject out cross-validation. We used the mask consisting of 16 clusters identified as different between groups as a 2^nd^ level mask in PRoNTo. Every single subject was classified correctly, achieving 100 % accuracy. Graph of prediction values and the associated density functions and weights map are shown in Fig. 2. While it is the combination of all weights that defines the model and individual contributions of voxels in the 2^nd^ level mask cannot be accessed directly, the weights map confirms that most discriminative voxels are the ones in the left temporal pole. Perfect accuracy implies that the b-values in these regions encode sufficient information for successful discrimination between the two groups.

Using only the top cluster, which showed most significant abnormality in patients according to the *t*-test, as a 2^nd^ level mask, the accuracy went down, but remained highly significant. Overall accuracy was 84.2 %, with specificity and sensitivity of 84.2 % (correctly classifying 16 out of 19 subjects in each group). Follow-up permutation testing (with 2000 repetitions) confirmed that both the overall accuracy (*p* = 0.005) and the class accuracies (*p* = 0.005; *p* = 0.005) were significantly above chance. Therefore, ACF b values from this part of brain alone contain sufficient information for significant discrimination between the two subject groups.

As a control, we repeated the classification using the entire grey matter mask as a 2^nd^ level mask. Gray matter mask was obtained from SPM8 and thresholded at 0.5. This resulted in reduced specificity of 53 %, not significantly greater than the one expected by chance. This confirms that we discarded most of irrelevant information when constraining the analyses to either of the two 2^nd^ level masks described above.

### Constrained dynamics are linked with locally dense/long-range sparse connectivity

**Fig. 3:**
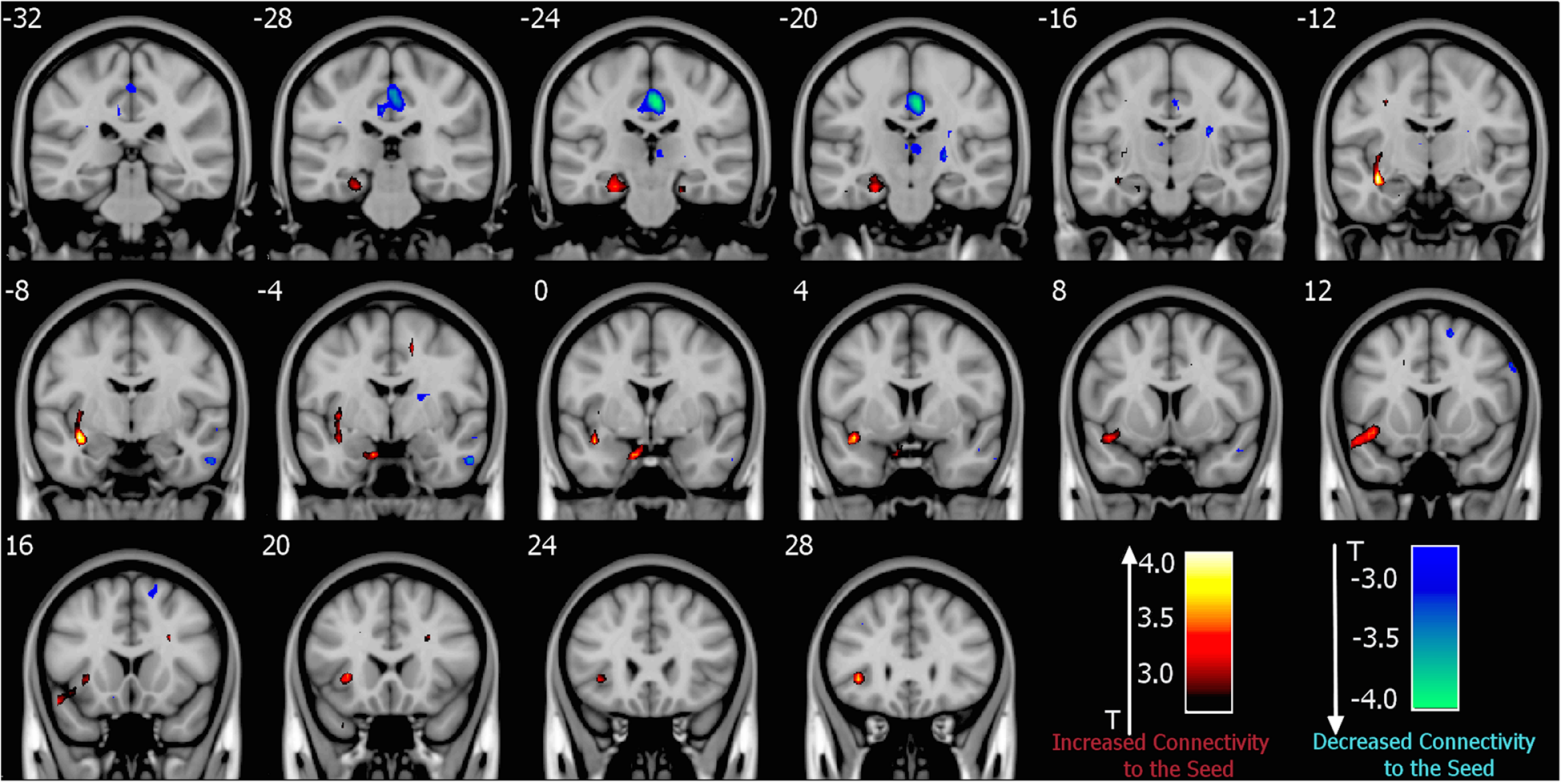
*Seed-to-voxel connectivity of the ACF-identified left temporal lobe cluster is significantly different in patients*. Relative to healthy controls, increases in connectivity are observed towards neighboring regions, while decreases are observed towards posterior cingulate cortex, thalamus, and the contralateral temporal lobe

Seed-to-voxel functional connectivity analysis revealed that LTLE patients show increase in connectivity of the most dynamically constrained cluster (ACF-identified cluster at MNI (−36 2–17)) with neighboring *left temporal* and *frontal regions*, as well as decreases in connectivity of this cluster with parts of *bilateral dorsal posterior cingulate cortex* (PCC), *contralateral inferior temporal gyrus*, and *contralateral thalamus*, when compared to healthy controls (*p* < 0.005 uncorrected; cluster extent > 10 voxels), as shown in Fig. 3. Table 3 contains a complete list of significant seed-to-voxel connectivity differences.

**Table 3:** List of regions showing significantly different functional connectivity to the ACF-identified left temporal pole cluster

	Left TLE > healthy controls			
	Peak MNI	Location description	Cluster extent	*t*-test (peak level T (p))
1	[−36, −10, −14]	L Temporopolar Area (BA 38), Hippocampus,	115	4.23 (7.70 × 10^−5^)
		Inferior Prefrontal Gyrus (BA 47),		
		Insular Cortex (BA 13)		
2	[−24, −25, −20]	L Perirhinal Cortex (BA 35),	40	3.34 (9.88 × 10^−4^)
		Parahippocampal Cortex (BA 36)		
3	[−36, 29, −2]	L Inferior Prefrontal Gyrus (BA 47)	27	3.67 (3.93 × 10^−4^)
4	[−33, −91, −2]	L Secondary Visual Cortex (BA 18)	19	3.35 (9.57 × 10^−4^)
5	[−27, −67, 31]	L Associative Visual Cortex (BA 19)	18	3.27 (1.20 × 10^−3^)
	Left TLE < Healthy Controls			
	Peak MNI	Location description	Cluster extent	*t*-test (peak level T (p))
1	[6, −22, 40]	Bilateral Dorsal Posterior Cingulate Cortex (BA 31), L Ventral Anterior Cingulate Cortex (BA 24)	97	−4.13 (1.04 × 10^−4^)
2	[54, −7, −29]	R Inferior Temporal Gyrus (BA 20)	12	−3.61 (4.58 × 10^−4^)
3	[15, 14, 58]	R Premotor Cortex (BA 6)	11	−3.36 (9.27 × 10^−4^)
4	[6, −22, 4]	R Thalamus	11	−3.11 (1.80 × 10^−3^)

Two-sample *t*-test was performed in CONN Toolbox (*p* < 0.005, k ≥ 10 voxels)

### Connectivity abnormalities correlate with epilepsy duration

In linking neural and clinical features, for the latter we focused on duration rather than seizure frequency since our patient sample did not provide significant variance in the latter. We found a significant positive linear correlation (Pearson’s r = 0.61, *p* = 0.005) between the mean connectivity of the cluster in PCC with the ACF-identified seed in left temporal pole and the duration of epilepsy. Therefore, while PCC exhibited overall decrease in connectivity with the seed in patients relative to controls, patients with longer duration of epilepsy had increased connectivity. On the other hand, for the cluster within the contralateral inferior temporal gyrus, there was a clear, but non-significant, trend towards negative correlation of this area’s connectivity with the seed and duration of epilepsy (Pearson’s r = −0.44, *p* = 0.060). For other areas that showed abnormal connectivity to the ACF-identified seed, connectivity with the seed did not exhibit linear relation to epilepsy duration.

### Connectivity abnormalities correlate with verbal memory task performance

**Fig. 4:**
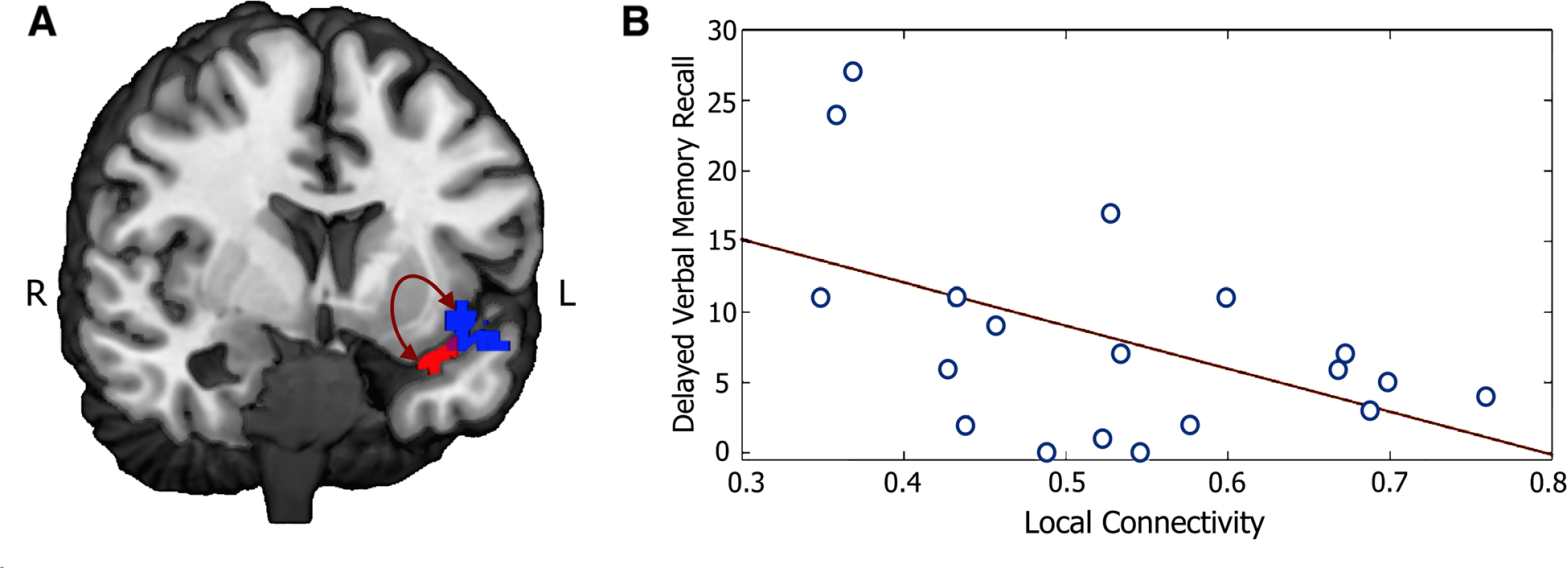
*Abnormalities in functional connectivity correlate with patients’ scores on the Logical Memory Delayed Recall task*. **a**: Connectivity of the dysregulated left temporal pole cluster (red) is increased with the neighboring temporopolar/insular/hippocampal region (blue) in patients compared to controls (Table **3**, Cluster 1). **b**: The strength of this local connection correlates negatively with patients’ performance on the verbal memory task (r = −0.50, *p* = 0.030, *N* = 19)

We found that disrupted local connectivity of the left temporal lobe ACF cluster correlated negatively with performance on the LM-DR task (Pearson’s r = −0.50, *p* = 0.03, Fig. 4); that is, patients who performed poorly on the verbal memory task exhibited higher local connectivity relative to those who did well. Greater severity of clinical symptoms was thus associated with higher local connectivity.

### Clusters with constrained dynamics form a less efficient network in patients

Global efficiency of the 16-node network was significantly decreased in patients compared to controls, with one-sided 2-sample *t*-test *p* < 0.05 when varying over costs of 0.20, 0.25, and 0.3 (corresponding to 20, 25, and 30 % of strongest connections kept; *p* = 0.011, *p* = 0.043, and *p* = 0.020, respectively). By thresholding at a certain cost we are in effect fixing the average degree of the network, so that the differences in graph measures presumably reflect changes in network topology. Nevertheless, it is important to note that there is no reliable and fully unbiased way of comparing networks, and that thresholding in this manner may convert non-significant values into edges for networks with low overall connectivity, and discard a number of significant connections for networks with high overall connectivity [60]. We did not find significant between-group differences in other graph metrics tested.

## Discussion

We found that patients with left TLE show slower time-series autocorrelation decay times in the left anterior temporal pole (proximal to the presumed epileptogenic zone) relative to decay times from the same region in healthy controls, indicating loss of complexity and more constrained functional dynamics. Decay time of the ACF discriminated successfully between patients and healthy controls on a subject-by-subjects basis in a purely data-driven manner, achieving accuracy of 100 % in our sample of *N* = 19, and therefore showing potential for future validation as a neurodiagnostic tool for localization of epileptic foci.

Changes in BOLD time series complexity were accompanied by changes in local and global connectivity, with increased connectivity with neighboring temporo-frontal areas, and decreased connectivity with regions of the default mode network (DMN) and the contralateral temporal lobe. This result agrees with recent findings that complexity covaries with local connectivity [61] as well as with recent ICA-based findings that MTLE specific networks (which include temporal poles) show increased connectivity in patients, whereas the control-specific network (including thalamus and anterior cingulate cortex) shows decreased connectivity in patients [15].

Complexity of low frequency BOLD fluctuations has been shown to correlate significantly with local connectivity as measured by regional homogeneity (ReHo) [61], implying that increased power spectrum scale invariance β (or, analogously, decreased autocorrelation function b values) may be a reflection of enhanced local synchronization. In addition, recent findings suggest that, relative to controls, unilateral MTLE patients show significantly increased regional homogeneity in the *ipsilateral parahippocampal gyrus*, but also in the *midbrain*, *insula*, *corpus callosum*, *bilateral sensorimotor cortex*, and *frontoparietal subcortical structures* [62]. This is in agreement with our findings of decreased ACF b values in a cluster within the *left temporal pole* and increased seed-to-voxel connectivity of this cluster towards neighboring areas, as well as with abnormal complexity found in the *inferior frontal gyrus*, *bilateral inferior parietal and postcentral area*, and *bilateral insula*.

The literature often reports structural changes in MTLE, which is most often accompanied by hippocampal sclerosis (HS), but also by (predominantly) ipsilateral atrophy of temporal pole and other temporolimbic structures [63]. It is clinically well established that mesial temporal sclerosis may extend throughout the temporal lobe, and involve the cortex and the white matter, thus leading to extensive temporal lobe atrophy. Increased diffusion rate and decreased anisotropy have been observed in the epileptic focus, of TLE patients with unilateral HS [13, 14]. Increased diffusion rate may be attributed to neuronal necrosis, gliosis, and expanded extracellular space, while the reduction in anisotropy may come from a loss of ordered structure, myelin degradation, and lowered cell density. Structural changes are often non-localizing, and even non-lateralizing, including altered diffusion properties in the *contralateral temporal and inferior frontal lobes* [14] and widespread significant neocortical thinning in the *sensorimotor cortex* [64]. Recurrent seizures and structural degeneration may lead to changes in functional connectivity as well: repair mechanisms (such as MMP-9) that degrade the matrix, or metabolic changes that impact glucose utilization, may facilitate local connections while preventing long-range connections.

Our recent simulation studies show that power spectrum scale invariance varies as a function of both input type (excitatory versus inhibitory) and input density, with greater density of inhibitory or lower density of excitatory inputs producing constrained (less complex, more persistent) dynamics [48]. Although epilepsy is often thought of as a hyper-excitatory disorder, this may not necessarily hold true during interictal periods (for example, it has been postulated that ictal epileptic neurophysiological activity can trigger local area neuronal network inhibition in attempt to stabilize the local neuronal network function [65]); constrained dynamics are, in fact, consistent with hubs that contain more inhibitory connections [48]. Furthermore, it has been postulated that epileptogenesis may involve not just the creation of a hyperexcitable state, but also the existence of high connectivity state and non-Markovian recurrent loops [66], in agreement with our finding that epileptogenic regions show higher local connectivity and exhibit longer time series memory (slower autocorrelation decay). The *posterior cingulate cortex* (PCC) is a pivotal hub for integration and mediation of information in the brain [67]. It has been implicated in a range of functions, and shown to be a part of the *Default Mode Network* (DMN) and *Dorsal Attention Network*. It has strong reciprocal connections to *mesial temporal lobe* memory-related structures [68]. Our ACF-identified cluster showed decreases in connectivity with PCC, in agreement with a couple of recent studies showing diminished connectivity between PCC and bilateral mesial temporal structures in left MTLE patients [21, 69]. In addition, our findings suggest that there is a linear relationship between left temporal pole connectivity with PCC and epilepsy duration, over which the connection is restored over time. However, this restoration seems to be accompanied by aggravated decrease in connectivity with the opposite temporal lobe.

The Wechsler Memory Scale is one of the most commonly used memory tests in patients with epilepsy, and is often a part of standard pre-surgical evaluation. Patients with temporal lobe epilepsy have been found to have lower scores when compared to healthy controls, but the tests on their own have been unable to lateralize temporal epilepsy successfully [70, 71]. We found that local increase in connectivity of the dynamically constrained cluster in patients correlated negatively with scores on the Logical Memory Delayed Recall task suggesting, as expected, that more severe symptoms were associated with higher local connectivity. Therefore, local connectivity of the left temporal lobe ACF-identified region was not only increased in patients compared to controls, but was also associated with severity of verbal memory impairment within the patient population. This is not surprising considering that the disconnected cluster included parts of affected hippocampus, a structure known to be heavily involved in memory processing.

### Limitations and future directions

The current gold standard for localization of focal regions includes identification of an epileptogenic zone on intracranial EEG recordings combined with postoperative seizure freedom following its resection. Due to invasive nature of intracranial recordings and the extensive temporal duration required to establish seizure freedom with confidence, data of such nature are limited. In this study, we utilized data from nineteen patients with concordant clinical findings, including results of long-term video EEG monitoring and structural MRI abnormalities confirmed by an experienced neurologist. While this group serves as a benchmark for the application of the technique developed here, and comparisons of individual ACF b maps to the mean control map point to clinically relevant region in 100 % of tested subjects, future work will address its applicability in subjects with other forms of epilepsy, ideally with the epileptogenic zone confirmed via invasive recordings. The method presented here is an alternative to multimodal methods such as EEG-fMRI, which has the advantage of eliminating the electrophysiological setups that are incompatible with some MR head-coils, avoids extensive set-up times, is completely fMRI data-driven (avoiding assumptions with respect to the shape of the hemodynamic response function and modeling of epileptic events), and utilizes only ~ seven minutes of resting state data without the necessity of active patient participation. Our aim is to provide a first step towards a non-invasive method that reliably detects focal regions in patients with drug resistant cryptogenic epilepsy.

## Conclusions

We developed techniques for the interictal identification of epileptic foci through complexity and network analyses of fMRI time-series. In a completely non-invasive and data-driven manner, based on complexity values calculated from resting-state fMRI images alone, we were able to achieve 100 % accuracy in distinguishing between 19 healthy controls and 19 epileptic patients with well-defined left temporal epileptic foci. Our method has shown high sensitivity and specificity in localizing focal points, while providing additional information about the underlying dynamics of epileptic brains. Since the method does not explicitly depend on existence of MRI-detectable structural abnormalities, it could eventually be applied to patients with epileptic foci that are inadequately defined or poorly localized based on current state of the art neuroimaging techniques. Functional MRI analytical methods that target network dynamics therefore hold promise in providing novel and clinically valuable tools for the identification and resection of epileptogenic foci.
